# Matrix Metalloproteases from Adipose Tissue-Derived Stromal Cells Are Spatiotemporally Regulated by Hydrogel Mechanics in a 3D Microenvironment

**DOI:** 10.3390/bioengineering9080340

**Published:** 2022-07-26

**Authors:** Francisco Drusso Martinez-Garcia, Joris Anton van Dongen, Janette Kay Burgess, Martin Conrad Harmsen

**Affiliations:** 1 Department of Pathology and Medical Biology, University Medical Center Groningen, University of Groningen, 9713 GZ Groningen, The Netherlands; f.d.martinez.garcia@umcg.nl (F.D.M.-G.); j.k.burgess@umcg.nl (J.K.B.); 2 W.J. Kolff Research Institute, University Medical Center Groningen, University of Groningen, 9713 GZ Groningen, The Netherlands; 3 Department of Plastic Surgery, University Medical Center Utrecht, 3584 CX Utrecht, The Netherlands; jorisavandongen@gmail.com; 4 Groningen Research Institute for Asthma and COPD (GRIAC), University Medical Center Groningen, University of Groningen, 9713 GZ Groningen, The Netherlands

**Keywords:** adipose tissue-derived stromal cells, matrix metalloproteases, hydrogels, GelMA, stiffness, stress relaxation

## Abstract

Adipose tissue-derived stromal cells (ASCs) are of interest in tissue engineering and regenerative medicine (TERM) due to their easy acquisition, multipotency, and secretion of a host of factors that promote regeneration. Retention of ASCs in or around lesions is poor following direct administration. Therefore, for TERM applications, ASCs can be ‘immobilized’ via their incorporation into hydrogels such as gelatine methacryloyl (GelMA). Tweaking GelMA concentration is a common approach to approximate the mechanical properties found in organs or tissues that need repair. Distinct hydrogel mechanics influence the ability of a cell to spread, migrate, proliferate, and secrete trophic factors. Mesenchymal cells such as ASCs are potent remodellers of the extracellular matrix (ECM). Not only do ASCs deposit components, they also secrete matrix metalloproteases (MMPs) which degrade ECM. In this work, we investigated if GelMA polymer concentration influenced the expression of active MMPs by ASCs. In addition, MMPs’ presence was interrogated with regard to ASCs morphology and changes in hydrogel ultrastructure. For this, immortalised ASCs were embedded in 5%, 10%, and 15% (*w*/*v*) GelMA hydrogels, photopolymerised and cultured for 14 d. Zymography in situ indicated that MMPs had a variable, hydrogel concentration-dependent influence on ASCs-secreted MMPs. In 5% GelMA, ASCs showed a high and sustained expression of MMPs, while, in 10% and 15% GelMA, such expression was almost null. ASCs morphology based on F-actin staining showed that increasing GelMA concentrations inhibit their spreading. Scanning electron microscopy (SEM) showed that hydrogel ultrastructure in terms of pore density, pore size, and percentage porosity were not consistently influenced by cells. Interestingly, changes in ultrastructural parameters were detected also in cell-free materials, albeit without a clear trend. We conclude that hydrogel concentration and its underlying mechanics influenced MMP expression by ASCs. The exact MMPs that respond to these mechanical cues should be defined in follow-up experiments.

## 1. Introduction

Adipose tissue-derived stromal cells (ASCs) are plastic adherent cells that express surface markers common to mesenchymal stromal/stem cells (MSCs) [1,2,3]. ASCs are multipotent and able to differentiate into adipocytes, osteoblasts, chondrocytes, and other phenotypes [4]. ASCs acquisition via liposuction makes these readily accessible, abundant and, overall, more convenient than other MSCs’ sources, such as bone marrow [5]. Hence, ASCs are ideal candidates for clinical applications for the reasons before-mentioned. Such applications are commonly performed via systemic or local injections at the afflicted site [2,6,7,8,9,10]. However, these approaches face disadvantages such as limited cell retention and low cell viability at the targeted anatomical site [11,12,13].

ASCs can be combined with carrier biomaterials such as hydrogels to tackle the before-mentioned disadvantages [11,14,15,16]. Hydrogels are 3D polymer networks with high water-retaining capacities, formed by covalent and non-covalent bonds [17,18]. These polymer networks mimic ECM biophysical properties that are not possible to replicate by traditional 2D cultures [19,20]. The loading of ASCs into hydrogels, including those based on gelatine methacryloyl (GelMA) [21,22], is described in literature [18,23,24]. The combination of ASCs and GelMA has tissue engineering and regenerative medicine (TERM) potential [25,26,27,28]. Literature reports GelMA-ASCs’ combination for skin, bone, cartilage, and vascular networks in vitro formation within the TERM field [29,30,31]. GelMA stands as an inexpensive, rapidly manufacturing semi-synthetic material derived from collagen. GelMA possesses the advantages of natural-derived hydrogels: biocompatibility and biodegradability; and synthetic hydrogels: tuneable mechanical properties [21,32]. GelMA hydrogels are formed primarily via covalent bonds under UV/VIS light photopolymerisation. Such covalent crosslinks allow GelMA to retain shape fidelity under standard cell culture conditions [32,33,34].

Tweaking hydrogel concentrations is a common approach to approximate the mechanical properties of native tissues for TERM purposes [35,36,37]. When in 3D, cells sense the mechanics of their pericellular environment and elicit biological responses [38,39]. The expression of matrix metalloproteases (MMPs), among other enzymes, enable cells to degrade their surrounding environment, facilitating their spreading, migration, or proliferation, among others [40]. ASCs can alter the stiffness and viscoelasticity of GelMA hydrogels [41] through elusive mechanisms; however, this might relate to a combination of MMPs’ degradation and matrix deposition.

Thus, in this work, we investigated if the expression of ASCs-derived MMPs would be altered by hydrogel concentration and its related mechanics, namely elastic modulus (i.e., stiffness) and stress relaxation. We also investigated if MMPs expression could be reflected in hydrogel swelling ratio and ultrastructure changes after 14 days. We also evaluated ASCs’ morphology in histological stains. If GelMA-ASCs constructs are aimed for clinical use, their characterisation in vitro is relevant to know the end product before patient applications.

## 2. Methods

### 2.1. GelMA Synthesis

GelMA was synthesised by a one-pot method using medical grade gelatine type A, 99.8 kDa MW, 262 g Bloom (MedellaPro^®^ > 600 MW, Leverkusen, Germany). Gelatine (100 g) was dissolved in 1× Dulbecco’s phosphate-buffered saline (DPBS; BioWhittaker^®^, Walkersville, MD, USA) and 0.6 mL of Methacrylic anhydride (MAA; Sigma–Aldrich, Darmstadt, Germany) was added per gram of gelatine at 40 °C with gentle stirring for three hours. The solution was diluted in an equal volume of 1× DPBS, centrifuged at 2000× *g* for 5 min., and the supernatant decanted into a 14 kDa dialysis tube (Sigma–Aldrich, Darmstadt, Germany). The tube was dialysed for a week at 30 °C with demi-water that was replaced twice daily. Once the dialysis was completed, the solution was lyophilised in a Free Zone^®^ 2.5 Plus freeze dryer (Labconco Corporation, Kansas City, MO, USA) at −80 °C until dry. The degree of functionalisation (DoF) was determined through ^1^H-Nuclear Magnetic Resonance (^1^H-NMR), as described previously [41]. The GelMA working solution was prepared by dissolving the lyophilised GelMA powder in 1× DPBS at 5%, 10% and 15% (*w*/*v*) concentrations. All working solutions were mixed with the photoinitiator lithium phenyl-2,4,6-trimethylbenzoylphosphinate (LAP; Allevi Inc., Philadelphia, PA, USA) at a concentration of 0.5% (*w*/*v*) at 50 °C. Once dissolved, the working solutions were filter-sterilised with 0.2 µm polyethersulfone membrane filters (Corning^®^, Kaiserslautern, Germany) and stored in the dark at −20 °C until further use.

### 2.2. 3D Cell Culture

Immortalised ASCs (iADSC13) [42] were cultured in gelatine-coated T75 flasks (Corning^®^ Costar^®^, Darmstadt, Germany) in growth media composed of high glucose Dulbecco’s modified Eagle medium (DMEM; Lonza, Walkersville, MD, USA) containing 10% Fetal Bovine Serum (FBS; Sigma–Aldrich), 1% Penicillin-Streptomycin (Gibco™, Paisley, UK) and 2 mM L-glutamine (BioWhittaker^®^, Verviers, Belgium).

**Figure 1:**
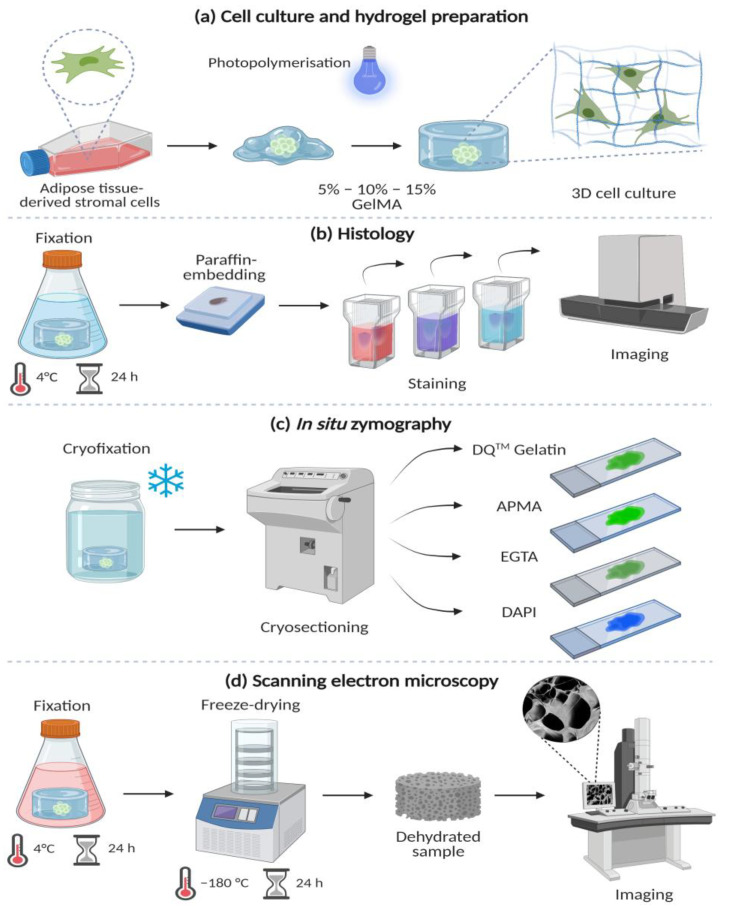
Methods: (**a**) Culture of adipose tissue-derived stromal cells (ASCs) and loading in 5%, 10%, and 15% gelatine methacryloyl (GelMA) hydrogels. (**b**) Histology stains after 4% formalin-fixed and paraffin-embedding. Sections (4 µm) were stained with hematoxylin and eosin (H&E), picrosirius red (PSR) and phalloidin/DAPI stains to observe the cellular morphology and distribution within the GelMA hydrogels. (**c**) In situ zymography to detect matrix metalloproteases (MMPs). Hydrogels were cryofixed (liquid nitrogen), cryosectioned (4 µm), and exposed to four different conditions: DQ^TM^ Gelatin, DQ^TM^ Gelatin/APMA (MMP activator) DQ^TM^ Gelatin/EGTA (MMP inhibitor) and DAPI alone. Cell-free material was also stained. (**d**) Scanning electron microscopy (SEM) of GelMA hydrogels after fixation with 2% paraformaldehyde and 2% glutaraldehyde followed by freeze-drying and visualised at 1000× magnification. Created with biorender.com (accessed on 18 July 2022).

All cells were harvested with 0.5% trypsin-EDTA (Sigma–Aldrich) after reaching 80% confluence and counted with an automated cell counter (Beckman Coulter™, Brea, CA, USA). Aliquots of 7 × 10^5^ ASCs were resuspended in 350 µL of either 5%, 10%, or 15% (*w*/*v*) GelMA working solutions and thawed at 37 °C. The GelMA-ASC suspensions were cast in 48-well plates (Corning^®^) and photopolymerised at 7 mW/cm^2^ for 5 min. with a UV/blue light lamp (405 nm). All gels, including cell-free (acellular) hydrogels, were cultured in growth media for 1 d, 7 d, and 14 d at 37 °C, 5% CO_2_ (Figure 1a). Only cells negative for *Mycoplasma* spp. from passages 19 to 22 were employed in these experiments.

### 2.3. ASCs Morphology

Cell-loaded hydrogels of 1 d, 7 d, and 14 d were fixed with 2% formalin for 24 h, followed by a series of ethanol dehydration and paraffin wax embedded (Figure 1b). Sections of 4 µm thickness were prepared with a microtome and mounted in microscopy slides StarFrost® (Waldemar Knittel, Braunschweig, Germany). All sections were conventionally deparaffinised in xylene and in a graded series of ethanol followed by hematoxylin and eosin (H&E) staining to visualise the ASC’s morphology. Sections were stained with Picrosirius red for 1 h in a single batch to reduce staining variability [43]. Staining with Texas Red™-X Phalloidin (Invitrogen™, Waltham, MA, USA) and 4′,6-diamidino-2-phenylindole (DAPI; Sigma–Aldrich) for 1 h allowed to visualise the cytoskeleton and the cell nuclei. All sections were imaged with Leica DM4000B fluorescent microscope (Leica Microsystems, Wetzlar, Germany) with the following filter light cubes: DAPI (λ_ex_ BP 340–380 nm/λ_em_ BP 450–490 nm) and TXR (λ_ex_ BP 540–580 nm/λ_em_ BP 593–668 nm) at 20× magnification.

### 2.4. In Situ Zymography

Cell-loaded and cell-free hydrogels were snap-frozen in liquid nitrogen and cryosectioned (4 µm thick) for experiments (Figure 1c). Evaluation of ASCs-derived MMPs was assessed using DQ™ Gelatin (EnzChek™ Molecular Probes, Eugene, OR, USA), which consists of gelatine densely labelled with fluorescein such that its fluorescence is auto-quenched. Upon proteolytic degradation of DQ™ Gelatin, the fluorescein is released and yields a bright green fluorescence signal locally [44]. Stock solutions were prepared as follows: 1 mg of DQ™ Gelatin and 5 mg of DAPI (Sigma–Aldrich) were individually diluted in 1 mL of DPBS. Then, 20 µL of DAPI stock solution were diluted in 50 mL of DPBS to a working concentration of 2 µg/mL (1:2500). DQ™ Gelatin was dissolved in the DAPI working solution (1:5). Additionally, the presence of inactive MMPs was detected by adding 4-aminophenylmercuric acetate (APMA) solution [3 mM/mL] at a 1:1 ratio to the DQ™ Gelatin working solution. Inhibition of MMPs was demonstrated by mixing the DQ™ Gelatin working solution with EGTA (20 mM/mL), a known MMP inhibitor [44]. The fluorescent dye mix (30 µL per section) was placed on top of the unfixed cryosections and incubated at room temperature (RT) for 1 h, protected from light. Cell-free hydrogels were also exposed to the before-mentioned conditions. After incubation, all stained cryosections were gently washed with DPBS before mounting in Citifluor™ AF1 (Electron Microscopy Solutions, Hatfield, PA, USA) and visualised with a Leica DM4000B fluorescent microscope with the following filter light cubes: FITC (λ_ex_ BP 460–500 nm/λ_em_ BP 512–542 nm) and DAPI (λ_ex_ BP 340–380 nm/λ_em_ BP 450–490 nm) at 20× magnification.

### 2.5. Swelling Ratio

Cell-loaded and cell-free GelMA hydrogels of 1 d, 7 d, and 14 d were carefully removed from the 48-well plates and weighed (*Ws*) on a scale (Sartorius Lab Instruments, Gottingen, Germany). All samples were freeze-dried for 24 h and weighed again to determine the dry weight (*Wd*). The swelling ratio was calculated as shown in Equation (1):

(1) $$Swelling\;ratio=\frac{Ws-Wd}{Wd}$$

### 2.6. Mechanical Properties

The 5%, 10%, and 15% GelMA hydrogels were swollen in 1 X DPBS for 24 h before mechanical testing on the Low-Load Compression Tester (LLCT) [41,45]. The LLCT is a non-destructive method that allows for determining the elastic and viscoelastic properties of hydrogels in terms of elastic modulus (i.e., stiffness) and stress relaxation (%), respectively. All hydrogels were quickly blotted to remove the excess water and mounted on standard microscopy glass slides. The specimens underwent uniaxial compression with a 2.5 mm plunger at RT. Hydrogels were compressed 20% (i.e., strain, ε = 0.2) of their original thickness at a strain rate (*ε*) deformation rate of 20%·s^−1^. The elastic modulus was determined during the first second of compression. For the stress relaxation percentage, the strain rate (*ε*) of 0.2 s^−1^ was kept constant for 50 s and calculated as the difference between *t* = 0 and *t* = 50 s. Data derived from three independent experiments were analysed with MatLab 2018 (MathWorks^®^ Inc., Natick, MA, USA).

### 2.7. Scanning Electron Microscopy

Cell-loaded and cell-free hydrogels of 1 d, 7 d, and 14 d were prepared, as described previously [46], by fixing with a 1:1 ratio mix of 1% paraformaldehyde and 1% formalin at 4 °C for 24 h. After, all samples were washed with DPBS and Milli-Q^®^ water, snap-frozen in liquid nitrogen, and freeze-dried for 24 h. The lyophilised hydrogels were mounted on top of 0.5″ pin stubs (Agar Scientific, Stansted, UK), placed inside a Leica EM SCD050 sputter coater device (Leica Microsystems B.V., Amsterdam, Netherlands), and rinsed and coated with Au-Pd one day before scanning electron microscopy (SEM) imaging. Hydrogels were visualised with Zeiss Supra 55 STEM (Carl Zeiss NTS GmbH, Oberkochen, Germany) at 2500× magnification, 3.0 kV, and Z = 40.0 mm (Figure 1d). A minimum of six random areas from three specimens per condition and timepoint were imaged and used to determine the pore density (i.e., number of pores), pore size (µm), and porosity percentage using ImageJ Ver 1.52p [47]. All images were transformed into 8-bit, and the number and size of particles (pores) were calculated.

### 2.8. Statistical Analysis

All statistical analyses were performed using GraphPad Prism v9.1.0 (GraphPad Company, San Diego, CA, USA). All data were searched for outliers using the robust regression and outlier removal (ROUT) test and analysed for normality using Shapiro–Wilk and D’Agostino and Pearson tests [48,49,50]. The swelling ratio pore size and porosity % data were analysed with a two-way analysis of variance (ANOVA) and Tukey’s post-test. Stiffness data were analysed with Brown Forsythe and Welch ANOVA and Dunnett’s T3 post-test. Stress relaxation data with one-way ANOVA and Tukey’s post-test. Data are presented as mean with standard deviation (SD) or median values with quartiles. *p* values below 0.05 were considered statistically significant.

## 3. Results

### 3.1. Cell Morphology in 3D

#### 3.1.1. H&E

**Figure 2:**
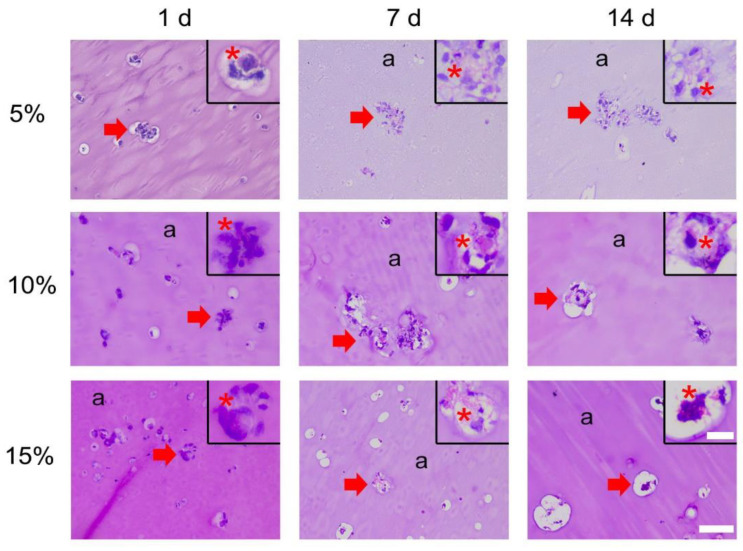
H&E stain of 5%, 10%, and 15% GelMA loaded with ASCs. Overview (Scale bar 100 µm) indicates the hydrogel (a) and the areas of clustered cells (red arrow). Such clustered areas are shown in the magnified view (Scale bar 25 µm), positive Hematoxylin (i.e., nuclei) seen as intense purple indicated with a red asterisk.

Cells were visible in all hydrogel sections with areas of clustered cells randomly distributed (Figure 2, red arrow). The staining intensity of the hydrogels increased with the GelMA concentration (a), but the nuclear material intensity remained similar, as shown in the magnified views (red asterisk). The most concentrated hydrogel (i.e., 15%) showed evidence of cutting artefacts (unidirectional cutting lines) due to the hardness of the material. The pericellular region showed a gap between cells and hydrogel in many cases.

#### 3.1.2. F-Actin

**Figure 3:**
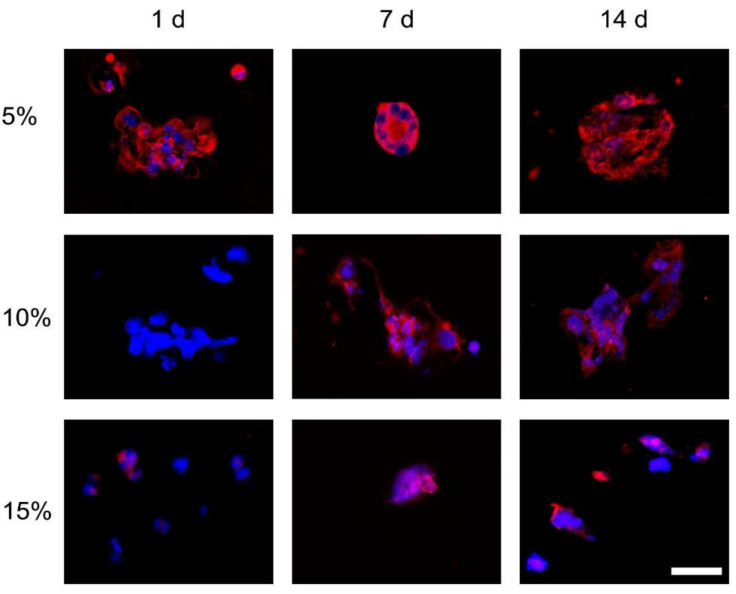
ASCs morphology in 5%, 10%, and 15% GelMA at 1 d, 7 d, and 14 d. Sections stained with DAPI and Phalloidin. Representative images are shown. Scale bar represents 25 µm, all fluoromicrographs have the same original magnification.

Morphology of ASCs differed across GelMA concentrations and time points (Figure 3). In 5% GelMA, phalloidin positive staining (red) demonstrated that cells spread at 1 d, 7 d, and 14 d. At 7 d, it became noticeable that certain cells were organised in spheroid-like structures. In 10% GelMA, the cell cytoskeleton was not noticeable at 1 d, but cell spreading was evident at 7 d and 14 d. Compared to the previous concentrations, cells in 15% GelMA remained rounded, indicating null spreading

#### 3.1.3. Picrosirius Red

**Figure 4:**
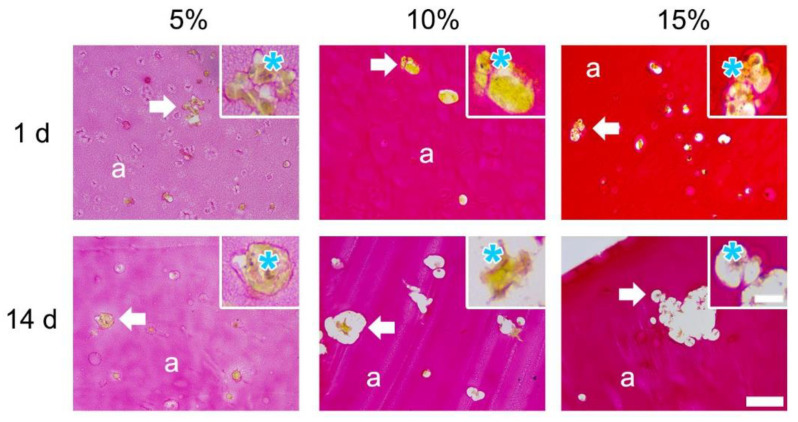
Picrosirius red stain of cell-loaded 5%, 10%, and 15% GelMA. Overview (Scale bar 100 µm) indicates the hydrogel (a) and the areas of clustered cells (white arrow). Such clustered areas are shown in the magnified view (Scale bar 25 µm); the cytoplasm of the cell and the nuclei are indicated with a cyan asterisk.

The staining time (1 h) was kept consistent for all GelMA concentrations and the staining intensity of PSR increased alongside hydrogel concentration comparable to the H&E staining (Figure 4). PSR allowed for visualisation of the cytoplasm (yellow) surrounding the cell nuclei (light purple). Cutting artefacts were present in 10% and 15% GelMA. Deposition of cell-derived collagen was visible in 5% GelMA as an increase in red stain surrounding the cells. In contrast, deposition of cell-derived collagen was not visible in 10% nor 15% GelMA due to the high degree of staining intensity of the hydrogel.

#### 3.1.4. MMPs Expression

**Figure 5:**
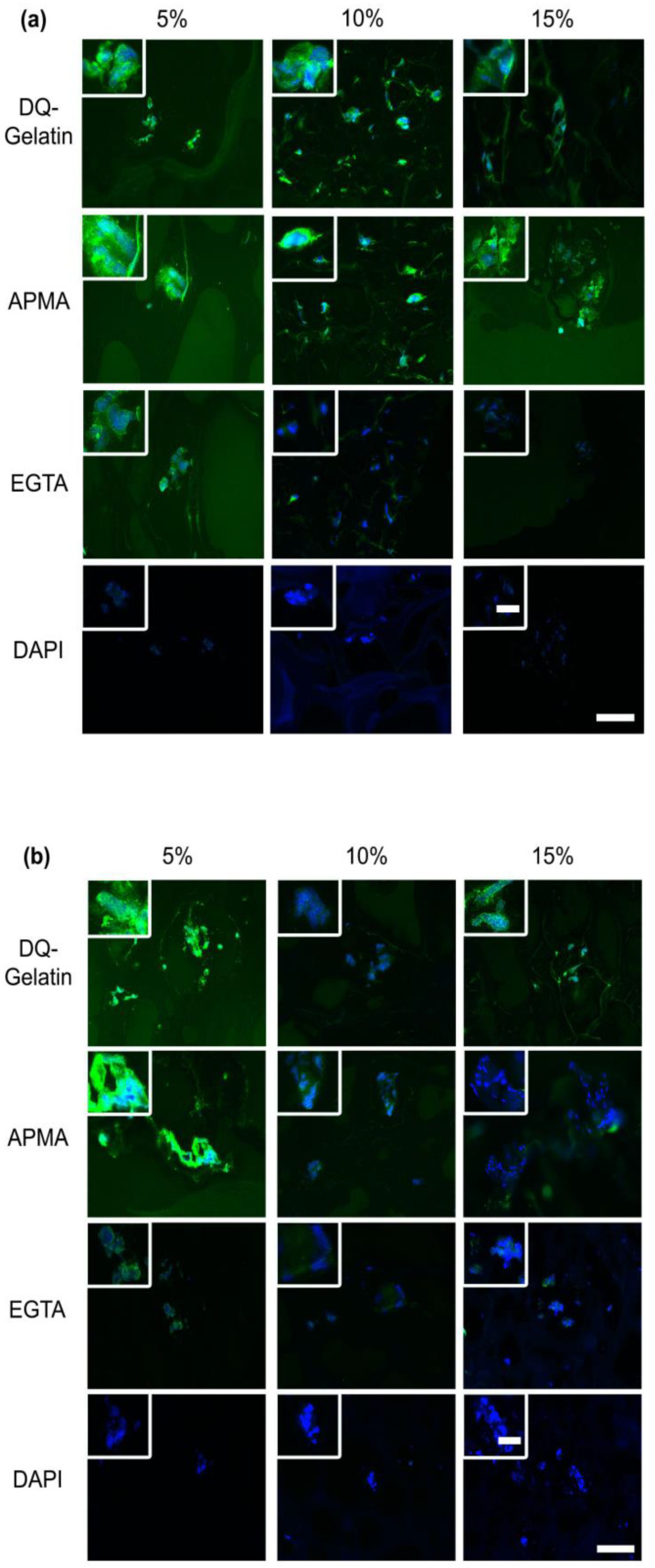
(**a**) Active ASC-secreted MMP in 5%, 10%, and 15% GelMA at 1 d. Cryosections stained with DQ-Gelatin, DQ-Gelatin/APMA (MMP activator), DQ-Gelatin/EGTA (MMP inhibitor), and DAPI only. Representative images are shown. Scale bars represent 10 µm (magnified view) and 50 µm (overview). (**b**) Active ASC-secreted MMP in 5%, 10%, and 15% GelMA at 14 d. Cryosections stained with DQ-Gelatin, DQ-Gelatin/APMA (MMP activator), DQ-Gelatin/EGTA (MMP inhibitor), and DAPI only. Representative images are shown. Scale bars represent 10 µm (magnified view) and 50 µm (Overview).

All ASCs secreted active MMPs irrespective of GelMA concentration after 1 d (Figure 5a). At 14 d, active MMPs were only detected in 5% GelMA (Figure 5b), while the presence of inactive MMPs was detected in 5%, 10%, and 15% GelMA. No MMPs were detected in cell-free hydrogels (Figure S1).

#### 3.1.5. Swelling Ratio

**Figure 6:**
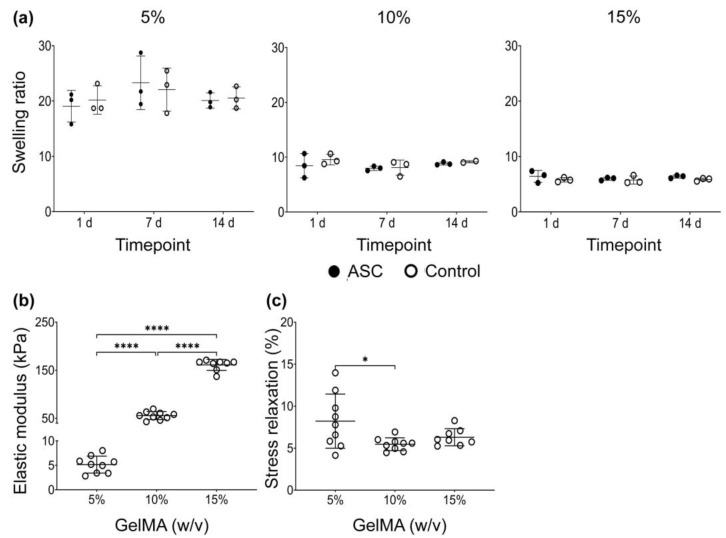
Swelling and mechanical properties of GelMA hydrogels: (**a**) Swelling ratio of cell-loaded (ASC) and cell-free (Control) GelMA hydrogels at 1 d, 7 d, and 14 d, calculated according to Equation (1). Data derived from three independent samples per timepoint. Data are presented as mean with standard deviation. No statistical differences were found according to two-way ANOVA and Tukey. (**b**) Elastic modulus (kPa) and (**c**) stress relaxation (%) of cell-free (Control) GelMA hydrogels after swelling for 24 h. Data derived from a minimum of three independent LLCT measurements per sample, from three independent experiments. Data are presented as mean with standard deviation. Differences in elastic modulus and stress relaxation according to Brown Forsythe and Welch ANOVA and Dunnett’s T3 post-test one-way ANOVA and Tukey’s post-test, respectively. *p* values are indicated * *p* < 0.05 and **** *p* < 0.0001.

The 5% GelMA had the highest swelling ratio of all concentrations, followed by 10% and 15%. ASCs did not change the GelMA swelling properties when compared across timepoints and cell-free material (Figure 6).

#### 3.1.6. Mechanical Properties

Increasing hydrogel concentration led to a significant, near-linear increase in elastic modulus (i.e., stiffness). The elastic modulus of 5%, 10%, and 15% GelMA hydrogels were 5.2 ± 1.7 kPa, 55.6 ± 8.4 kPa, and 161.1 ± 11.5 kPa, respectively. Regarding hydrogel viscoelasticity, the 5% GelMA (8.2 ± 3.2%) had a greater stress relaxation % than 10% GelMA (5.5 ± 0.8%) but not more than 15% GelMA (6.3 ± 1.0). No differences between 10% and 15% were found.

#### 3.1.7. Ultrastructure

**Figure 7:**
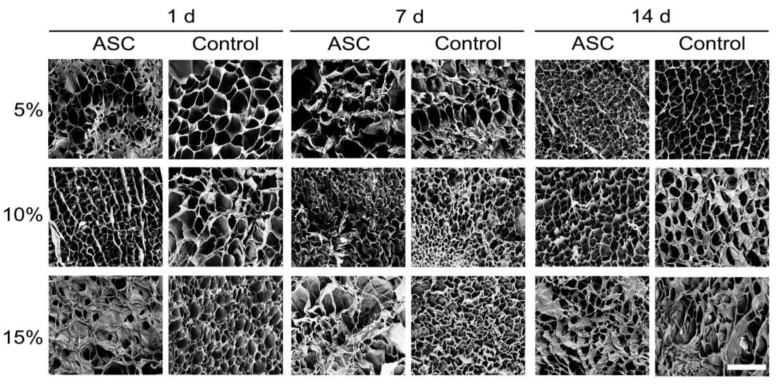
Surface ultrastructure of cell-loaded (ASC) and cell-free (Control) GelMA hydrogels according to scanning electron microscopy (SEM) at 1 d, 7 d, and 14 d. Representative images derived from three independent samples per timepoint, from a minimum of six random areas per hydrogel. Scale bar represents 40 µm. All micrographs have the same magnification.

**Figure 8:**
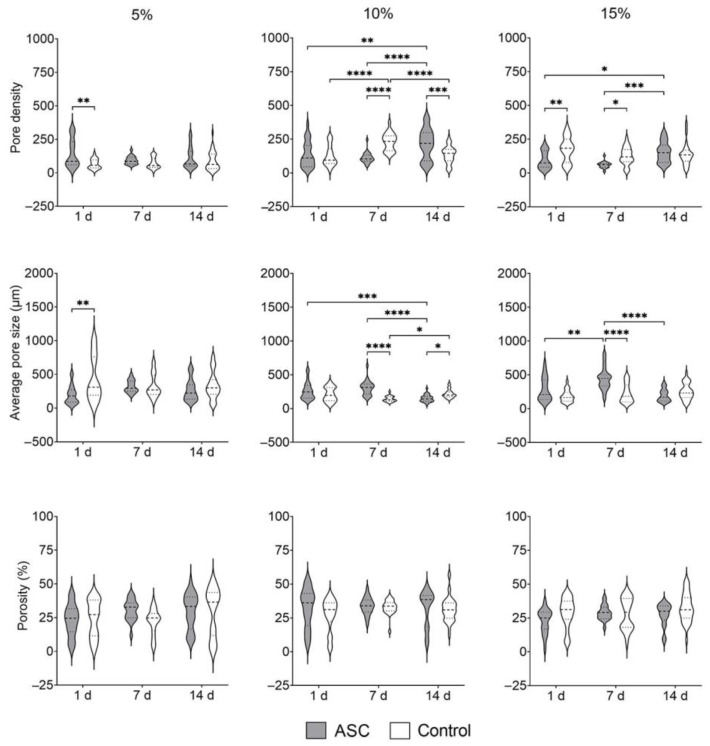
Analysis of GelMA architectural parameters based on SEM imaging of cell-loaded (ASC) and cell-free (Control) hydrogels. The pore density (i.e., number of pores), the average pore size (um), and the porosity % between cell-loaded (ASC) and cell-free (Control) hydrogels. Data derived from three independent samples per timepoint, from a minimum of five random areas per hydrogel. Data are presented as mean with standard deviation. *p* values are indicated * *p* < 0.05, ** *p* < 0.01, *** *p* < 0.001 and **** *p* < 0.0001 according to two-way ANOVA and Tukey.

The surface ultrastructure of cell-loaded and cell-free GelMA hydrogels was visualised with SEM (Figure 7). The analysis from SEM-derived images (Figure 8) showed that, in all GelMA concentrations, the percentage porosity remained unaffected by the presence of cells over time. Cell-loaded 5% GelMA had a greater pore density of smaller dimensions than cell-free hydrogels only at 1 d.

In 10% GelMA, cell-loaded material had a greater pore density after 14 d compared to 1 d and 7 d and its respective control. In contrast, the average pore size decreased after 14 d in culture. Interestingly, differences in pore density and average pore size were detected in cell-free 10% GelMA.

In 15% GelMA, pore density increased in cell-loaded hydrogels after 14 d. Compared to cell-loaded 1 d, the average pore size increased at 7 d but decreased at 14 d.

## 4. Discussion

Modulating hydrogel concentration is a common approach to tailor GelMA-ASCs’ hydrogels for TERM applications. In this work, we found that increasing hydrogel concentration led to an inhibition of active MMPs after 14 d in cell culture. The availability of active MMPs seems to be a spatiotemporal regulated process influenced by hydrogel concentration and mechanics. Such spatiotemporal regulation of MMPs is described in in vivo models with pathological conditions but is less studied in 3D in vitro models [51,52,53].

MMPs facilitate cell spreading by degrading the immediate microenvironment and reducing the mechanical constraint from the pericellular region [54]. Hence, it was expected that ASCs morphology would spread the most at higher MMPs expression; however, this only occurred in 5% GelMA. In 10% GelMA, ASCs spread after 14 d despite the null levels of MMPs detected. Thus, such spreading could be either derived by MMP-independent mechanisms [55,56] or by a lack of sustained MMPs secretion up to 14 d, hinting again towards a spatiotemporal process. In 15% GelMA, the presence of positively stained actin was minimal, showing no cell spreading, which is consistent with our previous report [41]. A high concentration of GelMA, i.e., 15%, might result in more binding of MMPs to gelatine, as compared to lower concentrations of GelMA [57]. In other words, fewer less MMPs will be free floating in the hydrogel. ASCs are known to also secrete tissue inhibitors of metalloproteases (TIMPs) which inhibit MMPs [45]. When a lower number of MMPs are free-floating, more of them might be relatively inhibited. This could potentially lead to lower spreading of ASCs in 15% GelMA.

ASCs’ spreading follows a clear trend regarding hydrogel concentration, where cell spreading is facilitated at lower polymer concentrations—where the elastic modulus is lower and exhibits higher stress relaxation. Thus, MMPs expression does not follow the same trend as spreading in terms of hydrogel concentration. Although GelMA has MMP degradation motifs, a lack of sustained expression suggests that MMPs’ release and activity are influenced by the pericellular mechanics. In our model, increasing hydrogel concentration led to a near-linear increase in the hydrogel elastic modulus. Reportedly, mechanics downregulated MMPs through TIMPs, albeit in 2D hydrogel models [58]. Previously reported [41], ASCs decreased GelMA stiffness after 14 d, regardless of hydrogel concentration. However, a decrease in stiffness might be insufficient to induce MMP activation. Time-dependent mechanics, such as stress relaxation, might facilitate MMP expression and activation [59]. In our study, 5% GelMA had a faster stress relaxation percentage than 10% and 15% GelMA. Such GelMA stress relaxation can be altered by ASCs in contrasting manners, changing from increasing (5%), sustaining (10%), or decreasing (15%) it [41]. Hence, ASCs-derived MMPs’ expression and activation seem to be predominantly modulated by hydrogel stress relaxation beyond sole stiffness, however further evidence is required to confirm this. Overall, GelMA hydrogels had a lower stress relaxation compared to non-covalent bound hydrogels previously characterised through LLCT [46,60].

The relation between hydrogel concentration and the inhibition of MMPs is of clinical relevance. GelMA hydrogels are well suited as carrier biomaterial to deliver ASCs on site of injection to decrease osteoarthritis or to heal osteochondral defects [61,62]. On one hand, a higher concentration of GelMA provides more stability and rigidness to the hydrogel. A stable construct is necessary in load-bearing joints such as the knee to prevent hydrogels from collapsing. When hydrogels collapse, ASCs will be released to the environment as single cells and tend to migrate from the site of injection. On the other hand, a higher concentration of GelMA appears to inhibit MMPs’ activity, resulting in no cell spreading. The question arises if these ASCs are still biologically active to improve the surrounding microenvironment, e.g., heal bony defects or decrease inflammation in joints.

Polymer degradation also impacts hydrogel mechanics, although most studies characterise such a phenomenon in cell-free materials [63,64,65]. Such approaches overlook cell-induced degradation and the subsequent impact on hydrogel’s 3D architecture and water-retaining capacities. Indirect methods to evaluate hydrogel degradation include swelling and ultrastructure analysis. Our data did not show any differences regarding the swelling ratio of hydrogels, and changes observed in ultrastructural parameters did not follow a clear trend either. The study of the ultrastructure parameters evaluated are highly dependent on a sample preparation method, which means the data is biased when utilising SEM [46,66,67]. Nevertheless, other authors concluded that 3T3 fibroblasts increase GelMA pore sizes based on SEM imaging [56]. Other imaging methods, such as second-harmonic generation, have shown a decrease in the pore sizes of cell-loaded collagen hydrogels, a contrasting finding [68].

Interestingly, cell-free GelMA also changed in terms of ultrastructure parameters across timepoints, and it is unclear if such changes are due to artefacts during specimen preparation for SEM, hydrogel contraction during culture, or additional changes in hydrogel conformation. Overall, data on the hydrogel structure in its native wet state is scarce and even less present when describing cell-loaded materials. We also recognise the additional limitations of our current work. For example, matrix deposition, such as ASCs-derived collagen, was not discernible in PSR stain and might require further histological assessments. Evidence indicates that hydrogel concentration influences MMPs’ expression, such as MMP-2 and MMP-9 [69]; however, we did not explore particular MMPs, and this remains to be investigated. Since the type of MMPs produced by cells is linked to their lineage [70,71], the ASCs phenotype in the different environments should be investigated. Additionally, SEM visualisation is limited to the hydrogel surface and not to the internal structure where most cells are located. Specimen preparation for SEM introduces biases due to the dehydration steps before-mentioned [72]. Clearly, novel methods are required to determine that the hydrogel architecture and ultrastructure do not rely on sample dehydration.

## 5. Conclusions

Active MMP expression by ASCs is a regulated spatiotemporal process influenced by hydrogel concentration and its underlying mechanics. Hence, we observe that an increased GelMA hydrogel stiffness inhibits MMPs activity in a 3D microenvironment. A more gradual trend was seen in terms of the function of ASCs, i.e., less spreading and collagen deposition observed in the highest concentration hydrogels.

## Data Availability

The datasets generated during and/or analysed during the current study are available from the corresponding author upon reasonable request.

## Supplementary Materials

The following supporting information can be downloaded at: https://www.mdpi.com/article/10.3390/bioengineering9080340/s1, Figure S1. Cryosections of cell-free 5%, 10%, and 15% GelMA at 1 d and 14 d. All cryosections stained with DQ-Gelatin, DQ-Gelatin/APMA (MMP activator), DQ-Gelatin/EGTA (MMP inhibitor), and DAPI alone. Representative images are shown. Scale bar represents 50 µm (overview).
