# Supplementary material for: Matrix Metalloproteases from Adipose Tissue-Derived Stromal Cells Are Spatiotemporally Regulated by Hydrogel Mechanics in a 3D Microenvironment
Source: Bioengineering (Basel). 2022 Jul 26;9(8):340. doi: 10.3390/bioengineering9080340 (PMC9332414; doi:10.3390/bioengineering9080340)
Supplement: Supplementary file 1 [file bioengineering-09-00340-s001.zip › bioengineering-1789106-supplementary.pdf]

Supplementary

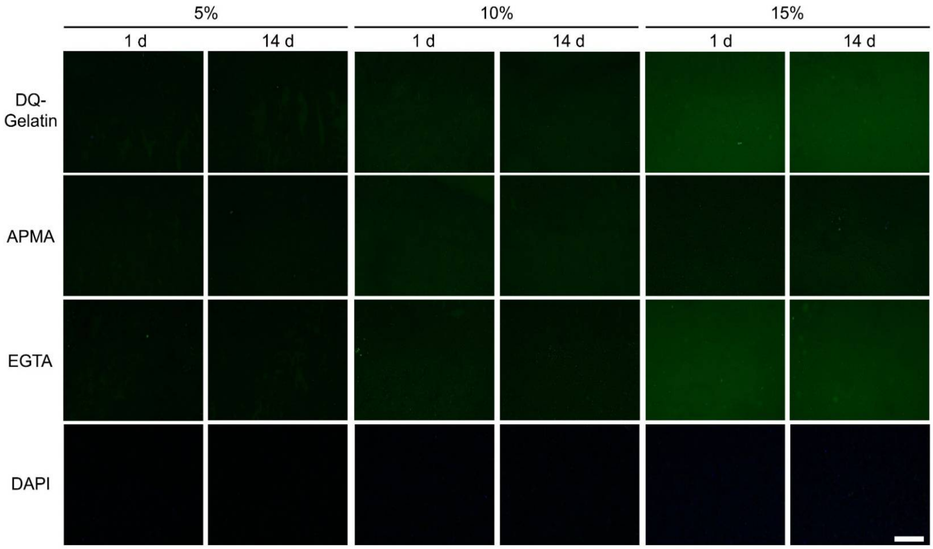

Figure S1. Cryosections of cell-free 5%, 10%, and 15% GelMA at 1 d and 14 d. All cryosections stained with DQ-Gelatin, DQ-Gelatin/APMA (MMP activator), DQ-Gelatin/EGTA (MMP inhibitor), and DAPI alone. Representative images are shown. Scale bar represents 50 μm (overview).
